# Cholesterol metabolism in the regulation of inflammatory responses

**DOI:** 10.3389/fphar.2023.1121819

**Published:** 2023-01-20

**Authors:** Rebekka Bauer, Bernhard Brüne, Tobias Schmid

**Affiliations:** ^1^ Institute of Biochemistry I, Faculty of Medicine, Goethe University Frankfurt, Frankfurt, Germany; ^2^ German Cancer Consortium (DKTK) Partner Site Frankfurt, Frankfurt, Germany; ^3^ Frankfurt Cancer Institute, Goethe University Frankfurt, Frankfurt, Germany; ^4^ Fraunhofer Institute for Translational Medicine and Pharmacology ITMP, Frankfurt, Germany

**Keywords:** cholesterol, inflammation, immunometabolism, SARS-CoV-2, COVID-19

## Abstract

The importance of biologically active lipid mediators, such as prostanoids, leukotrienes, and specialized pro-resolving mediators, in the regulation of inflammation is well established. While the relevance of cholesterol in the context of atherosclerosis is also widely accepted, the role of cholesterol and its biosynthetic precursors on inflammatory processes is less comprehensively described. In the present mini-review, we summarize the current understanding of the inflammation-regulatory properties of cholesterol and relevant biosynthetic intermediates taking into account the implications of different subcellular distributions. Finally, we discuss the inflammation-regulatory effect of cholesterol homeostasis in the context of SARS-CoV-2 infections.

## 1 Introduction

The high incidence of hypercholesteremia, i.e., pathologically elevated plasma cholesterol levels, remains a matter of great concern, as the concomitantly elevated levels of low-density lipoprotein (LDL) cholesterol pose a major risk factor for the development of cardiovascular diseases such as atherosclerosis (Tsao et al., 2022). In atherosclerosis, LDL-cholesterol is deposited in the arterial intima. Upon uptake of excessive amounts of modified, e.g., oxidized, LDL-cholesterol by macrophages, the latter develop a highly activated foam cell phenotype and, thus, contribute to the inflammatory character of atherosclerosis (Choudhury et al., 2005). Consequently, the use of cholesterol-lowering therapeutics, especially statins, i.e., 3-hydroxy-3-methylglutaryl-coenzyme A (HMG-CoA) reductase inhibitors, increased massively (Salami et al., 2017). The fact that upon inhibition of intracellular cholesterol production cells are able to cover their cholesterol demand by taking up cholesterol from the plasma (Gui et al., 2022), underscores the importance of maintaining intracellular cholesterol levels in a narrow, physiological range to facilitate the crucial functions of cholesterol in cellular membranes and as a precursor for various products (e.g., steroids, bile acids, vitamin D) (Tabas, 2002). Equally important, cells evolved efficient means to export cholesterol to avoid toxicity induced by excessive cholesterol levels (Song et al., 2021). As a side note, elevated cholesterol concentrations also play a pivotal role in the tumor microenvironment, where they not only support proliferation of tumor cells, but also attenuate anti-tumor immune responses. For details on the role of cholesterol homeostasis in tumor immunity see (Halimi and Farjadian, 2022).

With the recent advent of the concept of immunometabolism (Mathis and Shoelson, 2011), the tight integration of metabolic processes with immune responses gained increasing attention and led to propose that intracellular cholesterol dynamics and, similarly important, the cholesterol biosynthetic flux bear immune-regulatory properties (Fessler, 2016; O’Hagan et al., 2022). In this review, we provide a brief overview of the inflammation-regulatory functions of various cholesterol biosynthesis intermediates and discuss implications in the context of SARS-CoV-2.

## 2 Cholesterol and inflammation

### 2.1 Cholesterol homeostasis

All mammalian cells share essentially the same mechanisms to regulate their cholesterol content by a dynamic interplay between uptake, *de novo* synthesis, storage, and export. Cells take up cholesterol from the plasma, where it is transported predominantly packaged in LDL particles, *via* LDL receptor (LDLR)-mediated endocytosis (Brown and Goldstein, 1986). Upon release from the LDL particles within the endo-lysosomal compartment, cholesterol is distributed intracellularly to endoplasmic reticulum (ER) or plasma membrane by Niemann-Pick type C (NPC) 1 and 2 proteins (Pfeffer, 2019). In addition, cells can *de novo* synthesize cholesterol in a tightly regulated cascade involving more than 20 enzymes within three subcellular compartments (cytoplasm, ER, and peroxisomes) to satisfy their cholesterol demands (Charles et al., 2020). Due to its toxic properties, excess free intracellular cholesterol is esterified by acyl coenzyme A:cholesterol acyltransferase (ACAT) and either stored in lipid droplets or exported *via* ATP-binding cassette A1 (ABCA1) or ABCG1 and loaded onto high-density lipoprotein (HDL) (Yu and Tang, 2022).

**FIGURE 1:**
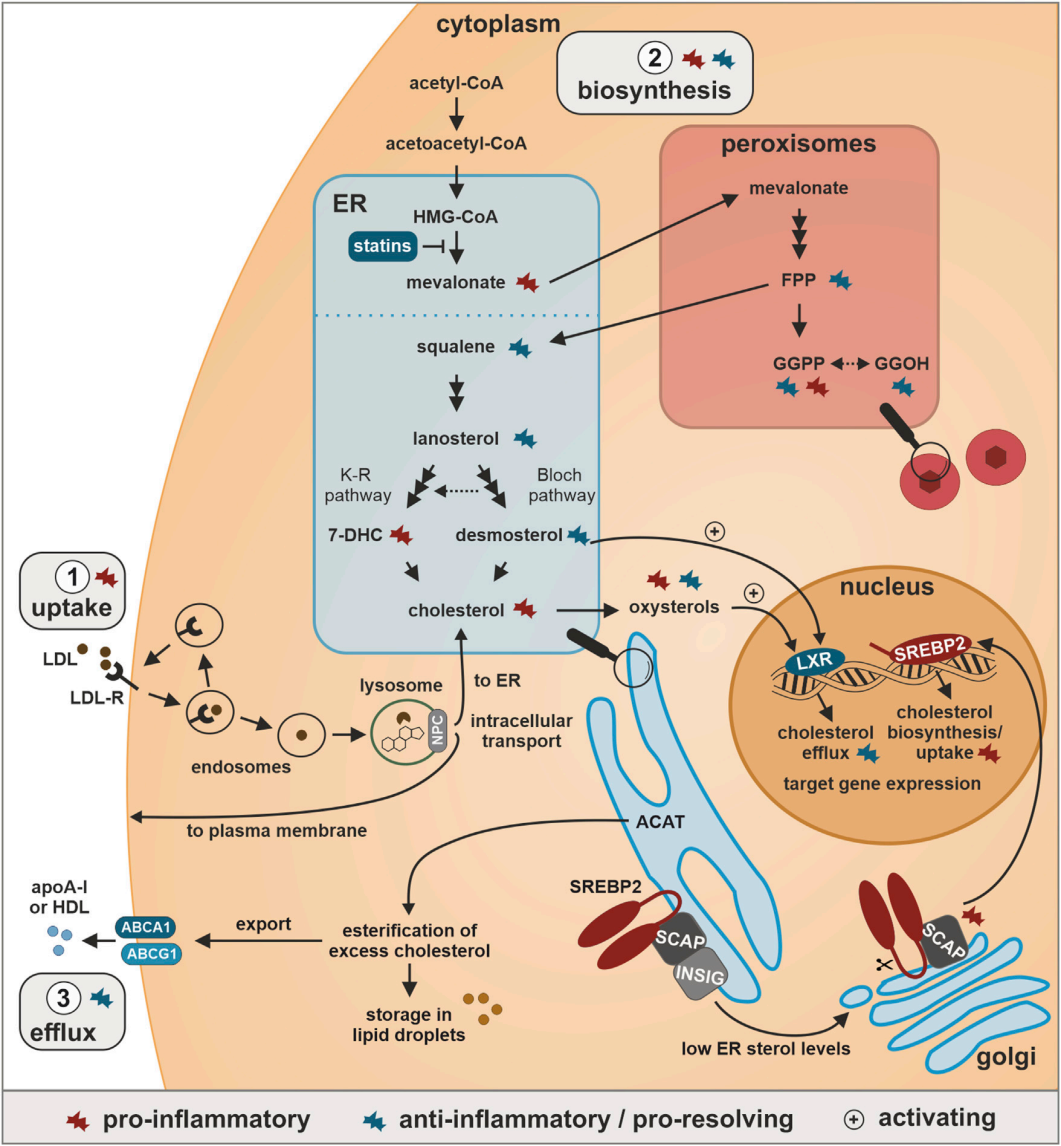
Cholesterol homeostasis and inflammatory regulation. 1. Uptake: Cholesterol is taken up bound to low-density lipoprotein (LDL) particles *via* LDL-receptor (LDL-R)-associated endocytosis, released from LDL upon fusion of endosomes with lysosomes, and distributed by Niemann-Pick type C (NPC) proteins to either the endoplasmic reticulum (ER) or the plasma membrane. 2. Biosynthesis: Cholesterol is synthesized starting from acetyl-CoA in the cytoplasm, converted *via* 3-hydroxy-3-methylglutaryl-coenzyme A (HMG-CoA) to mevalonate in the ER, further processed in peroxisomes to the isoprenoids farnesyl pyrophosphate (FPP) and geranylgeranyl pyrophosphate (GGPP), which can also be produced from the GGPP-precursor geranylgeraniol (GGOH). In the ER, two FPP molecules condensate to squalene, which in a multi-step process is processed to lanosterol, which is further metabolized in the parallel Bloch and Kandutsch-Russel (K-R) pathways to desmosterol and 7-dehydrocholesterol (7-DHC), respectively, both of which are direct precursors of cholesterol. ER cholesterol levels are sensed by sterol response element-binding protein 2 (SREBP2), which is bound by SREBP cleavage-activating protein (SCAP) and insulin-induced gene (INSIG) in the ER. Low ER cholesterol levels induce translocation of SCAP-bound SREBP2 to the Golgi, where it undergoes cleavage-dependent activation. The N-terminal SREBP2 fragment acts as transcription factor among others for cholesterol biosynthesis enzymes and LDL-R. 3. Efflux: Excess cholesterol in the ER is esterified by acyl coenzyme A:cholesterol acyltransferase (ACAT) and subsequently either stored in lipid droplets or exported *via* ATP-binding cassette A1 (ABCA1) or ABCG1 and loaded onto high-density lipoprotein (HDL) and apolipoprotein A-I (apoA-I) for retrograde transport to the liver. Elevated oxysterol and desmosterol levels further activate the transcription factor liver X receptor (LXR) to enhance the expression of cholesterol exporters.

The delicate cholesterol balance is largely controlled by two opposing transcription factors: Sterol response element-binding protein 2 (SREBP2), which, in response to cholesterol depletion at the ER, is escorted by SREBP cleavage-activating protein (SCAP) from ER to Golgi, where it is cleaved by site 1 protease (S1P) and S2P. The N-terminal transcription factor domain then localizes to the nucleus, and facilitates the expression of target genes, which are important for cholesterol biosynthesis and import (Sakai et al., 1996). In contrast, under elevated cholesterol conditions SREBP2 is retained at the ER, while oxysterols or desmosterol, a direct precursor of cholesterol, activate liver X receptors (LXRα/β) with subsequent induction of genes that reduce cholesterol uptake and promote cholesterol export (Janowski et al., 1996; Yang et al., 2006) (Figure 1). For further details, we refer the readers to the following comprehensive review (Luo et al., 2020).

### 2.2 Sterol synthesis and inflammatory responses

Cholesterol homeostasis and immune responses are tightly intertwined in a bidirectional manner. Since the present mini-review focuses on the effects of cholesterol (and cholesterol precursors) on inflammatory responses in innate immune cells, we refer the readers to excellent reviews for details on the reprogramming of cholesterol metabolism upon infections (Robertson and Ghazal, 2016; Lee and Bensinger, 2022).

A general reduction in the cholesterol biosynthetic flux was described to spontaneously induce interferon-stimulated genes (ISGs) (York et al., 2015), and to enhance anti-viral responses upon infections (Xiao et al., 2020). Moreover, SREBP2 was shown to directly bind and transcriptionally activate ISGs and further pro-inflammatory genes (Kusnadi et al., 2019). Independent of its SREBP2-activating function, the cholesterol sensor SCAP was also observed to connect cholesterol homeostasis to inflammation by shuttling interferon-regulatory factor 3 (IRF3) from ER to the golgi-localized stimulator of interferon genes (STING), thereby facilitating ISG induction upon infection (Chen et al., 2016), and by activation of the NLR family pyrin domain containing 3 (NLRP3) inflammasome (Guo et al., 2018). In the following sections, we will summarize the current understanding of the immunoregulatory functions of cholesterol and associated biosynthetic intermediates.

#### 2.2.1 Cholesterol

As exemplified by the pro-inflammatory character of foam cells laden with modified LDL-cholesterol in atherosclerosis (Tall and Yvan-Charvet, 2015), high cholesterol levels in innate immune cells are generally associated with pro-inflammatory functions. Along these lines, cholesterol crystals are recognized as NLRP3 inflammasome inducers in macrophages in atherosclerosis (Duewell et al., 2010).

In addition to total cellular cholesterol levels, its intracellular distribution appears critical for the inflammatory functions. For example, undisturbed cholesterol trafficking to the ER was required to enable activation of nuclear factor ‘kappa-light-chain-enhancer’ of activated B cells (NF-κB) and mitogen-activated protein kinase (MAPK) signaling, and consequently enhanced expression of the pro-inflammatory cytokines interleukin-6 (IL-6) and tumor necrosis factor-α (TNF-α) in response to excess amounts of free cholesterol (Li et al., 2005). Similarly, reducing ER cholesterol levels by either NPC1 inhibition or statin treatment abolished NLRP3 inflammasome activation, resulting in markedly lower IL-1β and IL-18 secretion. While activation of the NLRP3 inflammasome demanded intact shuttling of cholesterol to the ER, assembly and activation of the DNA sensing absent in melanoma 2 (AIM2) inflammasome by poly(deoxyadenylic-deoxythymidylic) acid (poly(dA:dT)) appeared insensitive to disturbances in cholesterol distribution (de la Roche et al., 2018). Interestingly, enhanced accumulation of cholesterol at the ER, brought about by experimental depletion of cholesterol-25-hydroxylase (Ch25h), resulted in mitochondrial (mt) dysfunction and mtDNA release and, thus, induced activation of the AIM2 inflammasome in response to lipopolysaccharide (LPS)-stimulation (Dang et al., 2017).

Furthermore, cholesterol, like other membrane lipids, affects the localization of various pattern-recognition receptors (PRRs) to specific organelles and/or membrane microdomains known as lipid rafts, which again is critical for the regulation of their activity. For further details, readers are referred to comprehensive reviews (Ruysschaert and Lonez, 2015; Köberlin et al., 2016). Therefore, it is not surprising that changes in intracellular cholesterol distribution were associated with altered toll-like receptor (TLR) sensitivity. For instance, increasing cholesterol loading in plasma membranes, by supplementation of methyl-β-cyclodextrin (CD)-complexed cholesterol, sufficed to initiate spontaneous TLR4 signaling in murine macrophages. In contrast, enhanced endosomal cholesterol accumulation, achieved by combined supplementation of acetylated LDL-cholesterol and inhibition of NPC1, induced TLR3 responses (Sun et al., 2009). A recent study further revealed that accumulation of free cholesterol in endosomes and lysosomes upon TLR4 activation is necessary for effective myeloid differentiation primary response 88 (MyD88)-dependent pro-inflammatory signaling in macrophages (Hayakawa et al., 2022).

In addition to uptake and *de novo* synthesis, cellular export of cholesterol by ABCA1/G1 transporters controls total and subcellular cholesterol levels, and consequently influences inflammatory responses. For example, intracellular cholesterol accumulation in ABCA1-deficient macrophages increased the expression of a broad range of inflammatory mediators upon LPS stimulation (Zhu et al., 2008). Mechanistically, elevated lipid raft cholesterol levels in ABCA1-deficient cells led to enhanced recruitment of TLRs to these membrane domains, thereby increasing the responsiveness to TLR agonists (Yvan-Charvet et al., 2008; Zhu et al., 2010). Cholesterol accumulation due to ABCA1/G1-deficiency was further shown to activate the NLRP3 inflammasome in dendritic cells, resulting in a systemic lupus erythematosus-like autoimmune phenotype in mice (Westerterp et al., 2017). This corroborates recent observations that miltefosine, an FDA-approved drug for the treatment of leishmaniasis, dampens NLRP3 inflammasome assembly and IL-1β release in macrophages by increasing ABCA1-mediated cholesterol efflux (Iacano et al., 2019).

Although cellular cholesterol export is largely associated with anti-inflammatory responses, the role of HDL remains controversial. In line with its function as a main plasma acceptor for cellular cholesterol, HDL was reported to provoke cholesterol export, thereby reducing cholesterol in lipid rafts and consequently attenuating TLR signaling (Cheng et al., 2012). HDL further induced the expression of activating transcription factor 3 (ATF3), a key transcriptional repressor of innate immune response genes, and, thus, downregulated the expression of TLR-induced pro-inflammatory cytokines (De Nardo et al., 2014). In contrast, HDL-associated apolipoprotein A-I (apoA-I) was shown to stimulate MyD88-dependent pro-inflammatory cytokine production in mouse macrophages (Smoak et al., 2010). Interestingly, while exporter-independent, lipid raft-disturbing depletion of cholesterol (e.g., in the presence of high HDL-apoA-I levels) was associated with pro-inflammatory effects, active, exporter-mediated efflux likely accounts for anti-inflammatory HDL effects (van der Vorst et al., 2017). Nevertheless, the majority of information, especially available *in vivo* findings, rather support the anti-inflammatory aspects of HDL (Fotakis et al., 2019).

Not surprisingly, LXR as the major regulator of ABCA1/G1 was also comprehensively analyzed in the context of inflammation. While the majority of reports described anti-inflammatory properties of LXR (Bilotta et al., 2020), others proposed potential pro-inflammatory functions of LXR as well. For instance, LXR was shown to activate hypoxia-inducible factor-1α (HIF-1α) and subsequent IL-1β secretion in human macrophages independent of its effects on ABCA1/G1 (Ménégaut et al., 2020). Furthermore, long-term activation of LXR was shown to potentiate subsequent LPS responses in human macrophages by increasing TLR4 signaling (Fontaine et al., 2007). Yet, considering the wide-ranging functions of LXR in lipid signaling, exceeding its mere role in cholesterol homeostasis (Wang and Tontonoz, 2018), contradictory findings regarding its impact on inflammatory responses are not entirely unexpected.

#### 2.2.2 Cholesterol biosynthesis intermediates

While the importance of cholesterol for innate immunity has been extensively studied, the role of specific intermediates generated during cholesterol biosynthesis for inflammatory reactions just emerges. In the following section, we will therefore briefly summarize the current understanding of the role of cholesterol precursors and oxysterols in the context of inflammation (Figure 1).

##### 2.2.2.1 Mevalonate

Mevalonate, the direct product of HMG-CoA reductase, is considered to have pro-inflammatory properties. In line, mevalonate accumulation enhanced a trained immunity phenotype induced by *β*-Glucan, and, consequently, contributed to elevated TNF-α and IL-6 responses after restimulation with LPS in human monocytes. Mevalonate-driven constitutive trained immunity was further proposed to contribute to recurrent episodes of inflammatory symptoms in patients suffering from the hyper immunoglobulin D syndrome (HIDS), which is characterized by mevalonate kinase deficiency (MKD) accompanied by elevated mevalonate levels (Bekkering et al., 2018). Interestingly, others proposed that the decrease in isoprenoids rather than the increase in mevalonate underlies the hyper-inflammatory syndrome in HIDS (Politiek and Waterham, 2021).

##### 2.2.2.2 Isoprenoids (farnesyl/geranylgeranyl pyrophosphate (FPP/GGPP))

The hydrophobicity-increasing prenylation of members of the Ras superfamily of small GTPases by the peroxisomally produced GGPP or FPP is essential for their functionality and localization, and, thus, directly affects cytoskeletal organization, receptor trafficking, and, consequently, numerous communication processes (Wang and Casey, 2016). Nevertheless, the role of isoprenoids in inflammation remains rather obscure. Some studies observed pro-inflammatory effects, due to enhanced type I or type II IFN signaling in response to GGPP (Veillard et al., 2006; Koike et al., 2021). In contrast, supplementation of the GGPP-precursor geranylgeraniol (GGOH) reduced pro-inflammatory cytokines after LPS stimulation *in vitro* and *in vivo* (Giriwono et al., 2013; Giriwono et al., 2019), suggestive for anti-inflammatory properties of GGPP. Accordingly, pharmacological inhibition of FPP synthase reduced FFP and GGPP levels, and concomitantly increased inflammatory cytokine expression in LPS-stimulated murine macrophages, which again could be reversed by the addition of GGOH (Tricarico et al., 2014). These findings substantiate the above-mentioned notion that the lack of isoprenoids might be accountable for hyper-inflammatory episodes in HIDS (Politiek and Waterham, 2021).

##### 2.2.2.3 Squalene

Cholesterol biosynthesis intermediates downstream of the isoprenoid pathway for the most part bear anti-inflammatory characteristics. In line, squalene, derived from the condensation of two FPP molecules, dampened NF-κB signaling and pro-inflammatory cytokine expression after LPS stimulation in monocytes and neutrophils as demonstrated by supplementation studies (Cárdeno et al., 2015).

##### 2.2.2.4 Lanosterol

Similarly, lanosterol accumulation, induced by pharmacological inhibition or knockdown of its metabolizing enzyme cytochrome P450 family 51 subfamily A member 1 (CYP51A1), reduced the expression of ISGs and pro-inflammatory cytokines after LPS treatment of murine macrophages (Araldi et al., 2017). Reduced lanosterol levels in mice transgenically overexpressing 3β-hydroxysterol Δ^24^-reductase (DHCR24), further correlated with progression of atherosclerosis, as well as with enhanced type I IFN responses and NLRP3-dependent inflammasome activation (Zhang et al., 2021). Of note, the authors characterized this pro-inflammatory phenotype mostly with respect to equally reduced desmosterol levels, yet, indicated that reduced lanosterol levels might also be involved.

##### 2.2.2.5 Desmosterol

Desmosterol is the direct precursor of cholesterol in the Bloch pathway. It was described to exert mostly anti-inflammatory functions by activating LXR. Accordingly, the above-described atherosclerosis-promoting phenotype in mice overexpressing DHCR24 in myeloid cells was proposed to result from the depletion of desmosterol, leading to impaired LXR activation and mitochondrial reactive oxygen species formation (Zhang et al., 2021). Along the same lines, DHCR24 inhibition and concomitant desmosterol accumulation, led to LXR-mediated synthesis of polyunsaturated fatty acids, which supported an anti-inflammatory and pro-resolving macrophage phenotype in a murine peritonitis model (Körner et al., 2019). Similarly, desmosterol-induced LXR activation in microglia contributed to inflammation resolution in multiple sclerosis (Berghoff et al., 2021). Interestingly, desmosterol accumulation was also observed in atherosclerotic foam cells, where it unexpectedly also suppressed inflammatory gene expression (Spann et al., 2012).

##### 2.2.2.6 7-Dehydrocholesterol (7-DHC)

In contrast, 7-DHC, the direct precursor of cholesterol in the Kandutsch-Russell pathway, was shown to exert pro-inflammatory functions. Specifically, elevated cellular 7-DHC levels, due to deficiency of 7-DHC reductase (DHCR7) or exogenous supplementation of 7-DHC, activated PI3K/AKT3, which contributed to increased IRF3 phosphorylation and type I IFN responses upon viral infections (Xiao et al., 2020).

##### 2.2.2.7 Oxysterols

Oxysterols are well-known for their anti-viral properties by influencing viral entry and replication *via* alterations of membrane compositions (Lembo et al., 2016; Foo et al., 2022). In addition, oxysterols were shown to induce pro- but also anti-inflammatory immune responses. Most of the anti-inflammatory effects of oxysterols, e.g., 25-hydroxycholesterol (25-HC), are mediated by activation of LXR (Ma and Nelson, 2019), but further LXR-independent functions were described. For instance, 25-HC accumulation restricted AIM2 inflammasome activation and IL-1β secretion in macrophages by reducing ER cholesterol levels and associated mitochondrial damage (Dang et al., 2017). In contrast, increased 25-HC levels enhanced pro-inflammatory responses *via* an activator protein-1 (AP-1)-dependent increase in TLR expression in macrophages in the context of viral infections (Gold et al., 2014). As the present mini-review aims to provide a quick overview of the role of various cholesterol biosynthesis intermediates in inflammation, readers specifically interested in the role of the more extensively studied oxysterols are referred to comprehensive reviews (Bah et al., 2017; de Freitas et al., 2022).

Conclusively, due to the complexity and highly dynamic regulation of cholesterol biosynthesis, which encompasses numerous feedback loops (Chen et al., 2019), characterizing the exact role of individual sterol precursors remains challenging as supplementation or interference with specific enzymes commonly affects a broad spectrum of other intermediates.

## 3 Implications for SARS-CoV-2 infections

Infections with severe acute respiratory syndrome coronavirus 2 (SARS-CoV-2) induce massive inflammatory responses. In severe cases of the resulting coronavirus disease 2019 (COVID-19), inflammatory events progress to a systemic inflammatory disease syndrome (SIRS), which correlates with poor disease prognosis (Jin et al., 2021). Importantly, while anti-viral, inflammatory responses are generally beneficial in early phases of the infection, development of overshooting systemic inflammatory responses, i.e., a cytokine storm, associated with excess production of pro-inflammatory cytokines, including type I and II IFNs, IL-1β, IL-6, IL-12, IL-18, and TNF-α, and chemokines, including C-X-C motif chemokine ligand 8 (CXCL8), CXCL9, CXCL10, CXCL11, C-C motif chemokine ligand 2 (CCL2), and CCL5, is rather detrimental (Coperchini et al., 2021). Interestingly, enhanced SREBP2 activity, indicative for elevated cellular cholesterol synthesis and biosynthetic flux, was found to correlate with disease severity in COVID-19 patients, and further directly associated with the development of a systemic cytokine storm (Lee et al., 2020). Paradoxically, lipoprotein-bound as well as total cholesterol levels in plasma appear to be reduced in COVID-19 patients, and low LDL- and HDL-cholesterol concentrations predict enhanced disease severity as well as mortality (Chidambaram et al., 2022). The apparent discrepancy between plasma cholesterol levels and SREBP2 activity in severe cases of COVID-19, might serve as an indicator for a generally disturbed cholesterol homeostasis. Presumably, this would result in an intracellular cholesterol overload, predicted to fuel pro-inflammatory responses (Dang and Cyster, 2019). This concept is corroborated by the recent finding that all classes of sterols (oxysterols and intermediates) were increased in extracellular vesicles (EVs) of COVID-19 patients during the hyper-inflammatory phase of the disease (Lam et al., 2021). Since EVs are not only important for intercellular communication (Grieco et al., 2021), but supposedly provide an accessible window into intracellular changes on a systemic level, these observations indeed hint towards increased SREBP2 activity. Considering the elevated levels of SREBP2-inhibitory 25-HC previously shown in response to viral infections (Reboldi et al., 2014), an increased cholesterol biosynthetic flux in the hyper-inflammatory phase of viral infections appears rather surprising. Nevertheless, the observation that the intermediates declined again in the EVs during the early resolution phase, agree with a dynamic response to increased 25-HC levels during the hyper-inflammatory phase (Lam et al., 2021). Taking the highly intertwined cholesterol dynamics into account, the success of therapeutic approaches interfering with cholesterol homeostasis in COVID-19 patients might be highly dependent on the exact disease stage. In fact, plasma EVs and selected cholesterol intermediates therein might be considered as an option to guide the appropriate timing of cholesterol-targeted therapeutic interventions. Of note, the HMG-CoA reductase inhibitor simvastatin was recently shown to reduce viral entry, but also to attenuate the expression of pro-inflammatory mediators in cells already infected by a virus (Teixeira et al., 2022). Furthermore, administration of 25-HC-containing nanovesicles effectively inhibited SREBP2 activity and reduced pro-inflammatory cytokines in COVID-19 patient derived immune cells (Kim et al., 2021).

Conclusively, we speculate that the cholesterol biosynthetic flux and/or levels of certain intermediates or cholesterol itself, as well as their intracellular distribution, might be directly involved in the systemic inflammatory progress of severe COVID-19 cases. This might provide novel opportunities for the development of molecular-targeted, disease stage-tailored interventions for COVID-19 patients. Specifically, while general cholesterol lowering measures bear protective potential during early stages of the disease by attenuating viral entry, the multi-facetted, partially contradictory effects of cholesterol and its biosynthetic intermediates likely require more sophisticated interventions strategies to prevent the detrimental hyper-inflammatory systemic course of the disease.
